# From the Frontline: Strengthening Surveillance and Response Capacities of the Rural Workforce in the Asia-Pacific Region. How Can Grass-Roots Implementation Research Help?

**DOI:** 10.3389/fpubh.2020.00507

**Published:** 2020-09-16

**Authors:** Sarah Larkins, Karen Carlisle, Humpress Harrington, David MacLaren, Etivina Lovo, Relmah Harrington, Lucsendar Fernandes Alves, Eric Rafai, Mere Delai, Maxine Whittaker

**Affiliations:** ^1^Anton Breinl Research Centre for Health Systems Strengthening, James Cook University, Townsville, QLD, Australia; ^2^Atoifi Health Research Group, Atoifi Adventist Hospital, Malaita, Solomon Islands; ^3^Fiji Institute of Pacific Health Research, College of Medicine, Nursing and Health Sciences, Fiji National University, Suva, Fiji; ^4^Menzies School of Health Research, Darwin, NT, Australia; ^5^World Health Organization, Dili, Timor-Leste; ^6^Ministry of Health and Medical Services, Suva, Fiji

**Keywords:** surveillance and response, communicable disease, implementation research, training, capacity strengthening, disease outbreak, Asia Pacific

## Abstract

Health systems in the Asia-Pacific region are poorly prepared for pandemic threats, particularly in rural/provincial areas. Yet future emerging infectious diseases are highly likely to emerge in these rural/provincial areas, due to high levels of contact between animals and humans (domestically and through agricultural activities), over-stretched and under-resourced health systems, notably within the health workforce, and a diverse array of socio-cultural determinants of health. In order to optimally implement health security measures at the frontline of health services where the people are served, it is vital to build capacity at the local district and facility level to adapt national and global guidelines to local contexts, including health systems, and community and socio-cultural realities. During 2017/18 James Cook University (JCU) facilitated an implementation research training program (funded by Australian Department of Foreign Affairs and Trade) for rural/provincial and regional health and biosecurity workers and managers from Fiji, Indonesia, Papua New Guinea (PNG), Solomon Islands and Timor-Leste. This training was designed so frontline health workers could learn research in their workplace, with no funding other than workplace resources, on topics relevant to health security in their local setting. The program, based upon the WHO-TDR Structured Operational Research and Training IniTiative (SORT-IT) consists of three blocks of teaching and a small, workplace-based research project. Over 50 projects by health workers including surveillance staff, laboratory managers, disease control officers, and border security staff included: analysis and mapping of surveillance data, infection control, IHR readiness, prevention/response and outbreak investigation. Policy briefs written by participants have informed local, provincial and national health managers, policy makers and development partners and provided on-the-ground recommendations for improved practice and training. These policy briefs reflected the socio-cultural, health system and disease-specific realities of each context. The information in the policy briefs can be used collectively to assess and strengthen health workforce capacity in rural/provincial areas. The capacity to use robust but simple research tools for formative and evaluative purposes provides sustainable capacity in the health system, particularly the rural health workforce. This capacity improves responses to infectious diseases threats and builds resilience into fragile health systems.

## Introduction

Emerging infectious diseases (EID) pose a serious threat in the Asia-Pacific Region, as do locally endemic communicable diseases (1, 2). Strong, resilient health systems and the ability of the local health and biosecurity workforce to recognize and respond to EIDs are key components of EID preparedness. This has been amply illustrated in recent months through the responses of low and middle income countries (LMICs) with already stretched and challenged health systems to the COVID-19 pandemic (3). A fit-for-purpose public health workforce, appropriately distributed, networked and with the required skill-sets is an essential part of detecting and responding to emerging and existing infectious diseases in the Asia-Pacific Region, while minimizing indirect deaths due to vaccine-preventable and other illnesses that tend to increase in a pandemic (3). Historically, however, insufficient investment has been made in the development and maintenance of skills-sets within the public health workforce, particularly those at the frontline in rural areas. Mobilizing frontline workers is a vital strategy for strengthening long-term capacity and responsiveness to detect and respond to EID threats while maintaining essential rural health services in the health systems of the Asia-Pacific Region.

Regions with a well-trained and fit-for-purpose health workforce enjoy both health and economic benefits (4). All too often, however, these benefits are concentrated in major urban centers, where there is access to further training, continuing professional development and other forms of support not extended to health and biosecurity workers outside major urban centers. In South Pacific countries most of the population live outside major urban centers (5). It is in rural areas where the risks of communicable disease outbreaks are highest, due to less access to health care, closer contact with animals (domestically and through agriculturally-based industries), population movement and personal contact patterns (for example, communal kava drinking in Fiji) and fewer personal protective resources (6). Delivery of services and surveillance and detection of outbreaks in small dispersed populations is more challenging and complicated to address due to maldistribution of health system resources, in particular the health workforce (7). Furthermore, the consequences of communicable disease spread are often greater in rural and regional areas, due to older and more vulnerable populations and less access to services (8).

Health and biosecurity workers in rural and remote contexts in LMICs are often extremely well-informed about local challenges to surveillance and response and local priorities for strategies to address these challenges. Many of these workers are involved in producing surveillance data such as Pacific Syndromic Surveillance System (PSSS) reports, but are not trained or supported to purposefully analyze the data or advocate for local solutions (9). Furthermore, there is a paradox that often less-experienced health and biosecurity workers are deployed to rural settings, which are viewed as less desirable, and these new graduates are provided with limited ongoing support or mentoring. For these reasons, supporting workers in rural and regional areas in the Asia Pacific Region to be active and empowered to improve primary health care systems and responses in their local context is likely to produce benefits and spin-off effects in terms of local health security as well as rural health workforce satisfaction and professionalization (and possibly retention).

One way to “activate” health and biosecurity workers with the skills and knowledge to respond to local surveillance and response challenges is through training in and conduct of implementation research projects. Implementation research (previously often called operational research) is the scientific study of the processes used to implement initiatives, and the contextual factors that may influence these processes (10). In the context of health, implementation research focuses on clinical and public health policies, programs, and practices, with the aim of identifying what does and does not work, and how and why this is the case in particular contexts. It also provides a structure through which to test implementation approaches.

In order to strengthen the research capacity of frontline surveillance and response staff, a series of implementation research workshops augmented with on-the-job work and mentoring was delivered to public health and biosecurity workers in Fiji, Solomon Islands, PNG, Timor-Leste and Eastern Indonesia in 2017–2018. These countries share common features of being island nations with widely distributed populations and extremely limited human resources for health (HRH). For example, Solomon Islands is classified as one of 57 countries deemed to have a critical shortage of health workers with a health worker density (physicians, nurses and midwives) of only 1.90 health workers per 1,000 population, well below the minimum WHO guidelines (of 2.3 per 1,000 population). In this country, there are only 87 doctors for the entire population of 550,000 (11, 12). Furthermore, few members of the stretched formal health workforce have any prior training in research or quality improvement (11).

In light of these contextual factors, the implementation research workshops adopted a “learning by doing” model whereby participants undertook a workplace-linked project on a surveillance and response priority issue identified in collaboration with local policy makers and in-country stakeholders (13, 14). Over 50 public health workers participated in the research training and conducted workplace-based projects, involving creating a protocol, obtaining ethics approval, and engaging in data analysis and dissemination of results. Projects covered a wide range of local priority areas including TB/Bovine TB, vaccine preventable diseases, vector-borne diseases and evaluation of surveillance and response systems.

**Figure 1:**
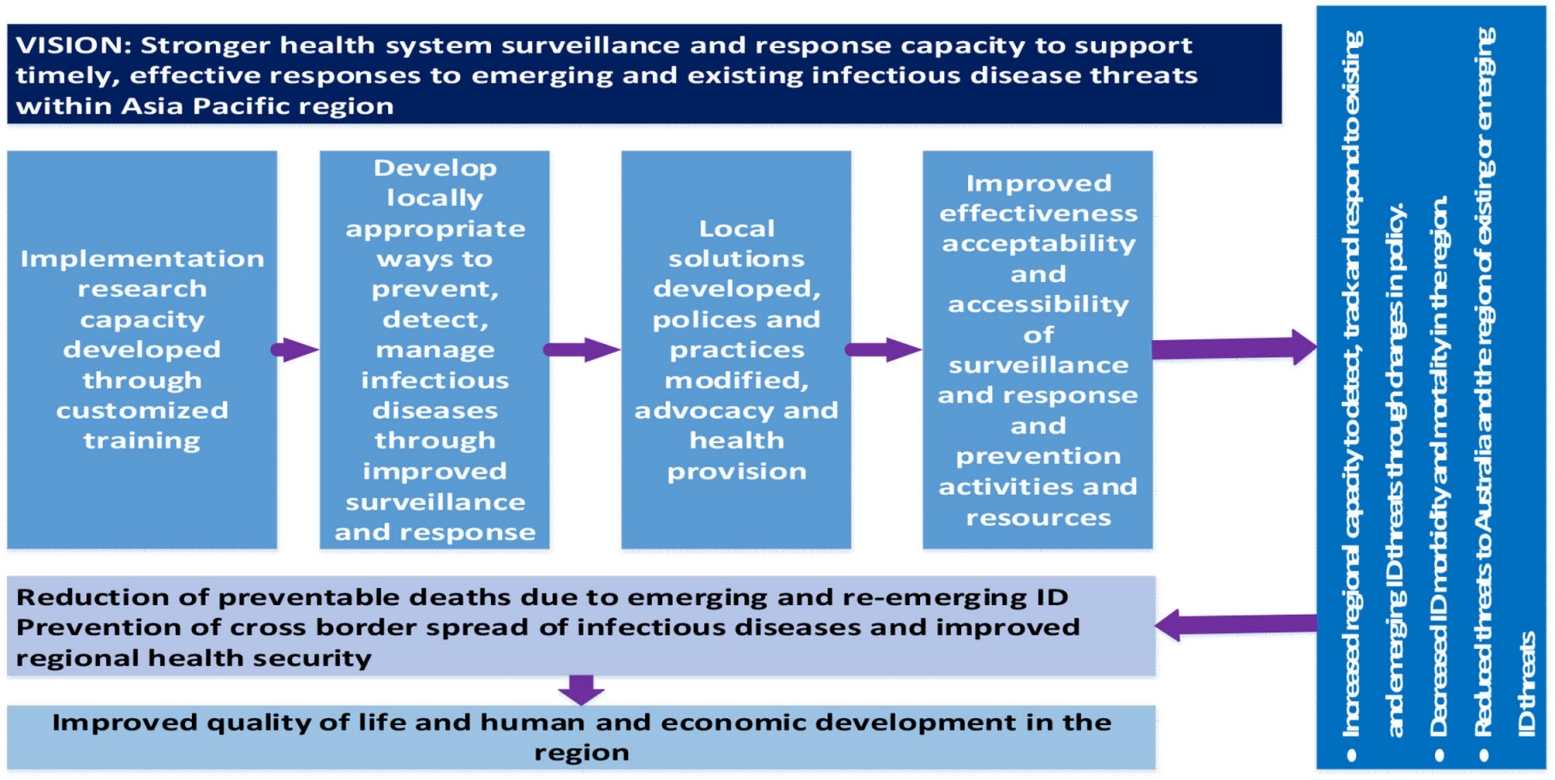
Program logic guiding capacity-strengthening activities of the Partners in Tropical Health project.

Ongoing engagement with key stakeholders in the project countries was prioritized throughout the project. This engagement included Ministries of Health, local universities, and non-government organizations (NGO), ensuring ongoing support and sustainability of this capacity-strengthening initiative. The implementation research training was part of a broader program of collaborative research projects based on surveillance and response in the Indo-Pacific region—the “Partners in Tropical Health” project. The program logic informing the capacity strengthening component of the project is presented below (Figure 1).

In this manuscript, we briefly report about the process and systems-wide outcomes of implementation research training for rural health and biosecurity workers in the Asia-Pacific, focused on surveillance and response to communicable disease threats. We then analyze the topics and health system components of completed projects to explore how interconnected outcomes can have an additive effect in supporting the rural health workforce in LMICs and strengthening health systems to provide both ongoing essential health care and responses necessary for health security. A companion paper has been developed wherein we explain the quantitative and qualitative changes in knowledge and confidence around implementation research among fellows in LMICs (Carlisle et al. under review). Lessons from this region may be of use to other LMICs as they consider strategies to strengthen the rural workforce to enhance preparedness for inevitable future novel infectious disease outbreaks.

## Methods

### Development of Local Partnerships to Agree on Priorities and Selection of Fellows

In-country meetings with stakeholders were held to develop surveillance and response-based priorities for research projects, and identify target participants and in-country mentors. The program was targeted at participants with at least 5 years of health workforce experience, although no previous research experience was expected or required. Following submission of an expression of interest, the selected frontline health workers (referred in this paper as Research Fellows) participated in three in-country workshops. Projects conducted by Research Fellows were designed to ensure that most research could be conducted in the workplace, complementing existing daily activities and ensuring that they were not lost to service delivery while conducting their projects. Forging relationships and fostering communication between health, biosecurity and agricultural surveillance and response staff within regions was an important secondary goal of the work.

### Customization and Delivery of the Implementation Research Training Program

The implementation research curriculum was based on the successful Structured Operational Research and Training IniTiative (SORT-IT) model (15). The SORT-IT model is designed to help low- and middle-income countries to improve their health systems through capacity-building and priority-driven research. Participants come from the health workforce and learn practical research skills through mentor-supported training and undertaking their own research projects.

**Figure 2:**
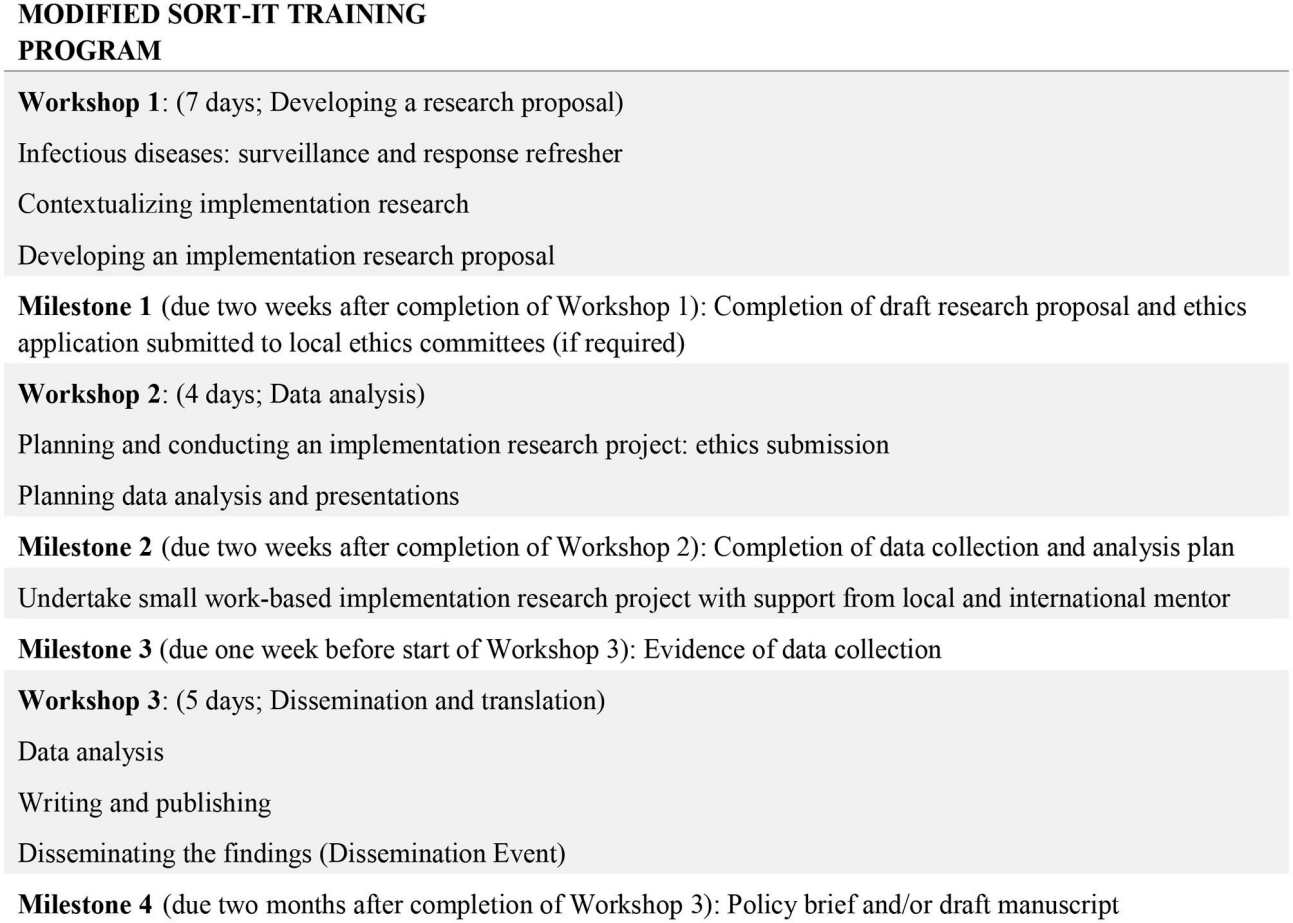
Structure and milestones of the modified SORT-iT program used in the Indo-Pacific for the Partners in Tropical Health project.

We customized the SORT-IT materials to ensure that the curriculum was regionally relevant based on in-country discussions with the partner countries, limited prior research exposure and the Tropical Partners focus on policy-relevant surveillance and response research (Figure 2). The delivered training consisted of a series of three face-to-face workshops, lasting 7, 4, and 5 days, respectively over a period of ~12 months, interspersed with periods of independent work supported by mentors. This training was repeated for cohorts based in Fiji, the Solomon Islands and eastern Indonesia.

The main modifications included: (a) expanding the focus on qualitative and mixed methods implementation research, (b) increasing the focus on the policy brief as a key research output, (c) changing the software used for quantitative analysis from Epi-data to Excel, and (d) reducing the expectation of prior research experience for selection into the training. This meant changing the original pre-requisite of Masters level qualifications, to no expectation of previous research experience or educational qualification. This opened eligibility to many frontline rural/provincial health workers who would otherwise be systematically excluded from such programs.

The participatory workshops ensured that Research Fellows (RFs) could fully develop their research experience and skills, with formal presentations, practical activities, small group discussions, time for writing, and presentations from the RFs themselves on their research proposals and results. Each RF was paired with in-country and international mentors for their project, in addition to ongoing support from the four project facilitators.

After the projects were completed (but before manuscripts had been completed), country-specific policy translation workshops were held. These sessions were well-attended by a wide range of intersectoral stakeholders, policy makers and leaders from government, health, higher education, biosecurity, animal health and livestock sectors. All RFs gave an oral presentation about their work and findings and lively cross-sectoral solution-focused discussions ensued.

### Evaluation of Outcomes

Evaluation of outcomes from the perspective of individual RFs in terms of changes in knowledge and confidence and satisfaction with the process was undertaken with pre and post-workshop questionnaires collecting quantitative and qualitative data. Findings were positive and statistically significant (Carlisle et al. under review). Evaluation of outcomes and effects is ongoing as RFs continue to finalize publications from their work. However, all RFs completed policy briefs and shared these with key decision-makers within their countries, leading to local and regional cross-sectoral knowledge exchange and awareness. To understand the broader outcomes and effect of the project findings, we undertook a process of mapping the thematic findings and then identified barriers and enablers to surveillance and response capacity identified by RFs against the six WHO health system building blocks (16), with the accepted addition of working with community. Initially undertaken by one facilitator (MW), this was then broadened to include all facilitators and the broader authorship.

From discussions following this mapping process, an explanatory framework was developed. This framework is designed to explore the associations between the rural health workforce, the community, and other health system components. Understanding these associations is a critical step to strengthen the capacity of the rural and remote health workforce to respond to communicable disease threats, and capitalize on the potential for context-aware priority research to inform policy and practice.

## Results

Fifty-three public health workers across five countries completed the implementation research training and conducted workforce-based projects (Table 1). Most of the participants were rural/provincial public health and biosecurity workers, but participants included primary health care and district health staff, laboratory staff, animal health officers, district hospital staff and provincial or national hospital staff as well as central health advisors. Overall 34 (64.2%) of RFs who completed the workshops were female. Table 1 includes a summary of prior educational attainment and occupational background of the RFs.

**Table 1:** Demographic characteristics of completed Research Fellows.

		**Number of participants (*N* = 53) *n* (%)**
**Country of residence**		
	Fiji	17 (32.1%)
	Solomon Islands	19 (36.4%)
	Papua New Guinea	5 (9.4%)
	Eastern Indonesia	11 (20.8%)
	Timor-Leste	1 (1.9%)
Sex	Female	34 (64.2%)
	Male	19 (35.8%)
Highest educational qualification	Doctoral degree	1 (1.9%)
	Master's degree	13 (24.5%)
	Bachelor degree	28 (52.8%)
	Diploma	11 (20.8%)
Occupational group	Surveillance and response worker (field/rural)	15 (28.3%)
	Nurse	13 (24.5%)
	Doctor	2 (3.8%)
	Biosecurity worker	3 (5.7%)
	Surveillance and response worker (provincial)	9 (17.0%)
	Lecturer/teacher	6 (11.3%)
	Laboratory Scientist	4 (7.5%)

Projects covered a wide range of local priority areas, including tuberculosis (TB) and bovine TB, vaccine preventable diseases, evaluation of surveillance and response systems, outbreak preparedness, and vector-borne diseases. The projects included analysis and mapping of surveillance data; infection control; readiness for implementation of the International Health Regulations (IHR); prevention and response and outbreak investigation.

### Foci of Studies From a Health Systems Perspective

Pleasingly, there was a high degree of correlation between the topics and foci chosen by RFs for their completed implementation research projects and the research and health system priorities that had been previously identified in meetings and workshops with health ministry, education and health sector stakeholders in each country. There was an extremely high level of satisfaction and knowledge-gain amongst participants (Carlisle et al. under review). The vast majority of RFs produced a policy brief and some have published their work, while others continue working toward publications for inclusion in the *Western Pacific Surveillance and Response Journal* and *Fiji Journal of Public Health*.

To synthesize project findings and extract the implications for rural health workers in LMICs, key barriers and enablers reported from the implementation research studies conducted by the RFs were mapped against the WHO health system building blocks (Table 2). The majority of projects had a focus on health information systems for surveillance and response, supporting the health workforce, community responses, service delivery and medical products and infrastructure. Smaller numbers of projects focused on health system governance and financing (Table 2).

**Table 2:** Project areas, health system building blocks, factors affecting health system capacity, and recommendations from Research Fellows.

**Building blocks (no. of projects)**	**Project areas of focus** **(CD including TB, malaria, dengue, zika, measles, diarrhea, leptospirosis, and meningococcal disease)**	**Factors affecting surveillance and response capacity**	**Synthesized fellow recommendations**
Health information systems (19 projects)	• Use of data for prediction, response, evaluation • Quality of data and adherence to protocols • Linked data (climate, geography, diseases)	• Under-reporting and double counting • Not using data for response/decision making/preparedness	• Strengthen surveillance and response systems, especially capacity of health workers to document and respond • Training for health and biosecurity workforce on recording, interpreting and sharing data
Workforce (11 projects)	• Clinical practices • Quality of training (and evaluation) • Surge capacity; barriers and enablers for workforce response • Knowledge and motivation • Health workforce numbers	• Inadequate knowledge and supervision • Motivation and adherence issues • Poor quality training • Inadequate staff numbers and exhaustion	• Invest in adequate health staff to respond to outbreaks (incl. surge) • Provide high quality ongoing training and professional development (incl. data recording) • Career pathways and support • Support training and PD in professionalism at all levels • QI processes around training and supervision
Community (8 projects)	• Health seeking behavior • Causes for delay • Lived experiences • Knowledge and behaviors re prevention (animal and human health)	• Limited health seeking behavior (related to knowledge and stigma) • Socio-economic and cultural determinants affecting ability to modify risk	• Target community education and health promotion to reduce stigma • Improve cultural safety of services • Consider role of community volunteers in surge capacity for education/health promotion
Medical products and infrastructure (6 projects)	• Antimicrobial resistance • Water, sanitation and waste disposal facilities • Supplies at health facilities	• Laboratory and health facilities ill–equipped with unreliable supplies • Lack of water and sanitation facilities at health facilities • Poor antibiotic stewardship • Limited surge capacity	• Review inventory and restocking systems • Ensure access to infrastructure required for safe care e.g., handwashing, waste management • Provision of basic equipment and maintenance
Service delivery (6 projects)	• Home based care • Community volunteers for dengue control • Improving immunization coverage • TB-DOTS • Net distribution	• Accessibility, affordability and acceptability issues • Inadequate health promotion • Underperforming community volunteers • Integration of volunteers with mainstream workforce	• Training and recognition of volunteers as important HRH • Budget to train family as partners in TB-DOTS • Free to user, distributed service provision • Mass immunization catch-up program
Governance (2 projects)	• Intersectoral collaboration (One Health) • International Health Regulations assessment	• Missing defined roles responsibilities, protocols, policies • Poor communications and inter-sectoral collaboration	• Standard operating procedures and policies for preparedness and response • Mechanisms for information sharing across sectors and levels of health system • Respond to feedback from HRH
Financing (1 project)	• Development assistance	• Important role of development assistance financing • Withdrawal/decline in financing leading to outbreak potentials	• Stable, ongoing programs of development assistance

*CD, Communicable Diseases; TB-DOTS, Tuberculosis - Directly Observed Treatment- Short-course; PD, Professional Development; QI, Quality Improvement; HRH, Human Resources for Health*.

The factors affecting health system capacity as identified by these implementation research projects were synthesized (Table 2). Key health systems factors identified by RFs were a lack of support for the rural health and biosecurity workforce, including poor quality training in priority areas, with inadequate knowledge and supervision. In turn, this reduced motivation and adherence to best practices. Inadequate staff numbers resulting in exhaustion and burn-out of the workforce also contributed, as did planning and information problems such as a lack of connection between identifying a problem and being able to produce a response. Limited surge capacity was an issue in terms of both workforce and supplies, and projects identified the important role of a volunteer workforce in filling gaps and assisting with service delivery, but also some challenges inherent in training, supporting and integrating this additional workforce. Recommendations to address workforce challenges included building training, support and career pathways for rural health professionals and strengthening engagement and support with capable volunteer members from rural villages to reinforce prevention strategies for infectious diseases at village level.

**Figure 3:**
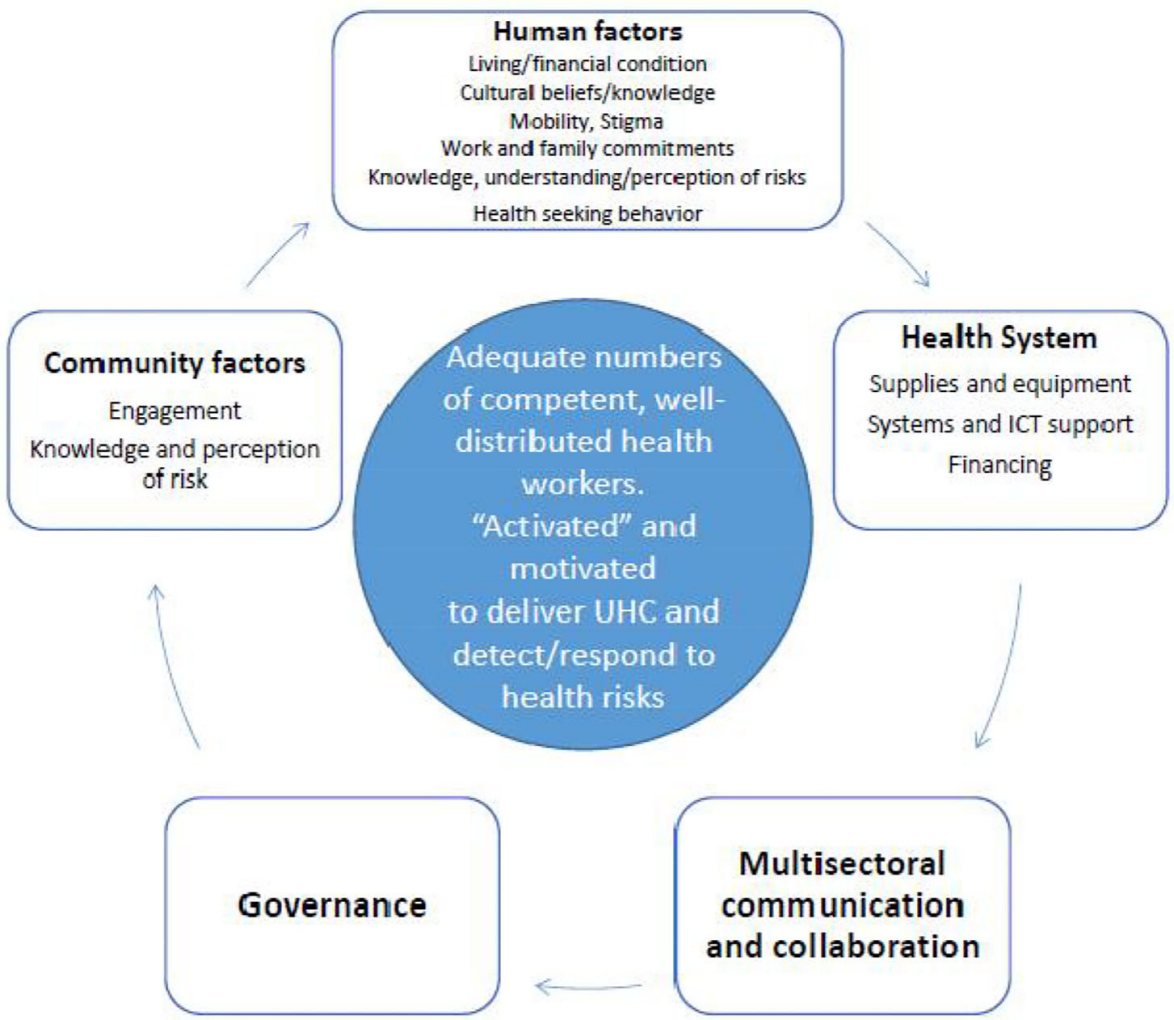
Interconnections for a stronger rural health workforce and better health security.

This synthesis of projects, findings and recommendations from RFs of our Partners in Tropical Health implementation research training was then used to develop an explanatory framework exploring the associations between the rural health workforce, the community, and other health system components that are vital to strengthen the capacity of the rural and remote health workforce to respond to communicable disease threats (Figure 3).

## Discussion

This Partners in Tropical Health implementation research training program successfully worked with rural health and biosecurity workers from five countries in the Asia-Pacific region, delivering a range of small but policy-relevant local research projects, and demonstrating successful rural health workforce development. The modified SORT-IT program appeared to be successful and well-received by participants, with significant gains in self-reported knowledge and confidence across a range of research skills (Carlisle et al. under review). The project topic areas, while organically developed, reflected local health sector priorities and covered a wide range of problematic local communicable diseases and health system building blocks. The individual project findings are being disseminated through in-country knowledge translation seminars, collections of policy briefs presented to local Ministries of Health, and peer-reviewed publications and presentations in locally-relevant journals. Whilst a limitation of this approach is the focus on small and simple research projects, perhaps limiting generalizability, this is far outweighed by the strength of generating locally relevant, contextually-informed solutions.

The explanatory framework synthesized from the analysis of these 53 projects reveals the strong inter-relationships between health system factors—primarily a competent, well-distributed health workforce, with individual and community drivers of good health, strong governance and inter-sectoral communication and information systems (Figure 3). These interconnected factors, so vital for health security are regularly being tested when responding to local/regional outbreaks of known infectious disease such as measles, polio, cholera, and dengue. Despite some success with the local outbreak detection of known infectious disease [aided in the Pacific by the embedded PSSS; (9)], retrospective analyses of responses to the International Health Regulations (IHR) indicate that many countries (notably in the WHO Africa and Western Pacific regions) have relatively low preparedness and operational readiness for responding to a global pandemic caused by a novel infectious agent. The current COVID-19 pandemic illustrates the additive burden for already stretched health systems dealing with outbreaks (e.g., dengue, measles) on an ongoing basis. The added threat of COVID-19 (or other EIDs) has the potential to overwhelm health systems (including the workforce) in parts of Asia and the Pacific (17).

There is a strong interconnection between health security (including the ability to respond to infectious disease threats) and overall health system capacity and resilience (18, 19). For example, responses to the Ebola-virus outbreak in East Africa showed the variability in responses and effectiveness depending on the underlying state of the health system and governance, and highlighted the inadequacy of existing health and medical research systems to understand and respond (20). Health systems need to have sufficient reserve to be able to recover after the shock of an infectious disease outbreak and still be able to provide ongoing care for people with chronic diseases, acute care for endemic infectious diseases and deliver appropriate preventive care (21).

What is striking are the parallels between our inductively-derived explanatory framework and those deductive frameworks used by the WHO and other large organizations to illustrate issues of pandemic preparedness and response more broadly (6, 22). Small, locally conducted implementation projects, conducted by locally embedded health and biosecurity workers with guidance in research methods (from international and local mentors) are able to deliver strong insights to local health sector leaders and ministry officials, while providing skills and confidence to distributed health workers, and at the same time linking with the local socio-cultural context and local determinants of health in this hyper-diverse region.

All of these issues are particularly critical in rural areas and low-income settings, where wide population dispersion and a scant health workforce add to the logistical difficulties in the delivery of health services (12). Health workforce challenges that are currently particular issues in the Asia-Pacific Region include: (a) absolute shortages; (b) maldistribution; (c) issues with governance, planning and support for the health workforce; (d) public sector working conditions; and (e) increasing global mobility of workers (19). However, there are many capable people in the rural settings who can be engaged—at village levels (e.g., unemployed youths with tertiary education achievements)—to strengthen data collection, and preparedness for the prevention of infectious diseases. These people communicate on a daily basis with other villagers to enforce prevention strategies for ID at village levels. During COVID-19, village women and young men were involved in training their own village members in hand washing, distancing, no sharing of cups in kava parties and other ways of contributing to prevention and preparedness. They do not have to be paid wages; they just do this work for the general good of the people of their village.

The rural and remote health and biosecurity workforce in LMICs is an underused and fragile resource in terms of preparedness and response to emerging infectious diseases and health security. The health workforce sits squarely at the intersection between delivering universal health coverage, having strong and resilient health systems, and global health security (18). Interventions focused on improving capacity to detect and respond to existing diseases that are locally prevalent and relevant can in addition help strengthen preparedness for a possible future EID pandemic (23). While the mechanism for this is as yet unclear, a plausible explanation is that a focus on local ongoing disease threats strengthens local engagement, recognizes the broader social and cultural determinants of health, and strengthens “soft” organizational capacity, such as communication and trust (24). The specificity of how this progresses in different locations needs to be guided by an openness to doing things in different ways in different contexts across the region. Adequate training, supplies, professional development and support are all important, as well as retention and recruitment initiatives. An additional benefit may follow in terms of synergies with several sustainable development goals (SDGs).

An additional factor that is less often mentioned is the importance of “activation” of the rural and remote health workforce. This can be thought of as analogous to empowerment, and is a vital factor in building health sector resilience (25). Developing an adequate knowledge and skills base to identify a problem, and design, implement and evaluate locally relevant and appropriate solutions to that health service problem is a critical competency–this is what this kind of IR training in a distributed style can deliver (26, 27).

## Conclusion

Policy briefs written by participants outlining results and recommendations from workplace-based projects have informed local, provincial and national health managers, policy makers and development partners. Recommendations have guided the development of improved health care practice and training. The capacity to use robust but simple research tools and processes for formative and evaluative purposes provides sustainable capacity to a distributed rural and remote health workforce for responding to infectious diseases threats. Scale-up of this approach may be warranted to strengthen surveillance and response. Strengthening the activation and motivation of these health professionals is an important element of this success.

## Data Availability Statement

The raw data supporting the conclusions of this article will be made available by the authors, without undue reservation.

## Ethics Statement

The studies involving human participants were reviewed and approved by James Cook University Human Research Ethics Committee. The patients/participants provided their written informed consent to participate in this study.

## Author Contributions

SL, MW, DM, and KC conceived this work and facilitated the workshop program together with HH and MD. MW conducted the initial synthesis of themes for Table 2, which was then reviewed and extended by other authors. SL wrote the first draft of this manuscript. All authors revised and commented on subsequent drafts, viewed and approved the final manuscript, and were involved with the program as facilitators, in-country industry partners, mentors or Fellows.

## Conflict of Interest

The authors declare that the research was conducted in the absence of any commercial or financial relationships that could be construed as a potential conflict of interest.
